# Editorial: Unravelling insect vector diversity: genetic and phenotypic insights from the Global South

**DOI:** 10.3389/finsc.2025.1734377

**Published:** 2025-11-24

**Authors:** Romina V. Piccinali, Ana L. Carbajal-de-la-Fuente, Julieta Nattero, Sebastián Pita, Carlos E. Almeida

**Affiliations:** 1 Laboratorio de Eco-Epidemiología, Departamento de Ecología, Genética y Evolución, Facultad de Ciencias Exactas y Naturales, Universidad de Buenos Aires, Ciudad Autónoma de Buenos Aires, Argentina; 2 Instituto de Ecología, Genética y Evolución de Buenos Aires (IEGEBA), CONICET-Universidad de Buenos Aires, Ciudad Autónoma de Buenos Aires, Argentina; 3 Centro Nacional de Diagnóstico e Investigación en Endemo-Epidemias (CeNDIE), Administración Nacional de Laboratorios e Institutos de Salud “Dr. Carlos Malbrán” (ANLIS), Ciudad Autónoma de Buenos Aires, Argentina; 4 Consejo Nacional de Investigaciones Científicas y Técnicas (CONICET), Ciudad Autónoma de Buenos Aires, Argentina; 5 Sección Genética Evolutiva, Facultad de Ciencias, Universidad de la República, Montevideo, Uruguay; 6 Laboratório Nacional e Internacional de Referência em Taxonomia de Triatomíneos, Instituto Oswaldo Cruz-FIOCRUZ, Rio de Janeiro, Brazil

**Keywords:** Global South, vector, genotype, phenotype, environment

The concept of the Global South emerged to promote collaboration among countries, mainly in the Southern Hemisphere, on political, economic, environmental, technical, and health issues (1). Among its most persistent health challenges are vector-borne diseases, whose dynamics are closely linked to the ecology, genetics, evolution, and diversity of their insect vectors (2–4). Many insect species function as biological and mechanical vectors, transmitting viruses, parasites and bacteria (5), and can even act as parasites themselves (6). Despite extensive control efforts targeting dengue, malaria, Zika and Chagas disease, among others, these infections continue to impose a considerable burden on public health systems and local communities (7, 8).

The remarkable environmental heterogeneity and high transmission rates make the Global South a key region for exploring relationships between genetic variation, phenotypic adaptation, and disease ecology. Strengthening our understanding of the genetic and phenotypic variability of vectors - such as mosquitoes, triatomines and flies - is essential to elucidate how these traits influence transmission patterns. This knowledge is particularly relevant in a world where climate change (9) urbanization (10), landscape transformations (11), and political or sanitary instability continuously challenge the implementation and sustainability of integrated vector control strategies (12).

## Genetic variation: endosymbionts, gene flow and taxonomy

Three papers in this Research Topic make use of genetic tools to shed light on taxonomic identification, gene flow, and the relationships between vectors and their endosymbionts. Bhuvaneshwaran et al. applied molecular tools to identify insects of medical and forensic importance. Their *cytochrome oxidase I* (COI) gene-based identification of maggots collected in Puducherry, India, enabled species-level diagnosis of flies, such as *Sarcophaga peregrina* and *Hemipyrellia ligurriens*. Molecular methods overcome the limitations of morphology-based identification in immature stages, providing diagnostic capability for public health investigations.

Chuchuy et al. examined the intracellular bacterium *Wolbachia pipientis*—a symbiont widely used in mosquito biocontrol—within *Aedes albopictus* populations from Argentina. The authors revealed striking patterns of strain-specific localization: both *wAlbA* and *wAlbB* coexist in ovarian tissues, but only *wAlbB* persists consistently in somatic tissues. These findings suggest that the tissue-specific *Wolbachia* distribution may be under natural selection, influencing vertical transmission and vector competence. By detailing *Wolbachia* density dynamics in natural populations, this work contributes valuable baseline data for optimizing *Wolbachia*-based control strategies in arbovirus-prone regions.

A complementary molecular approach is offered by Alqurashi et al., who analyzed *Ae. aegypti* populations from the urban centers of Jeddah and Jizan, Saudi Arabia. Using mitochondrial COI barcoding, they revealed substantial genetic divergence between the two populations, emphasizing the complex population structure of this key vector. The Jeddah population was genetically closer to those from Argentina and Australia, suggesting a shared ancestry or introduction route, while Jazan showed greater genetic diversity and affinities with mosquitoes from multiple regions, indicating a more diverse origin or higher gene flow. These results underscore the global connectivity of vector lineages and the importance of molecular surveillance in understanding invasion routes and potential gene flow across continents.

## Phenotypic variation: effects of landscape and hybridization over vector morphometrics

While molecular studies dissect genetic and endosymbiont diversity, a second group of contributions focuses on phenotypic variation and environmental adaptation, particularly in triatomine bugs—vectors of *Trypanosoma cruzi*, the euglenozoan responsible for Chagas disease. These papers illustrate how environmental pressures such as urbanization, habitat fragmentation and geographic gradients shape the morphology, dispersal potential, and ultimately, epidemiological relevance of vector populations. Fiad et al. investigated the effects of habitat fragmentation over flight-related traits in *Triatoma garciabesi* and *T. guasayana*. Using geometric morphometrics and landscape metrics, they found species-specific morphological responses to fragmentation. *T. garciabesi* displayed increased head asymmetry and narrower wings, while *T. guasayana* showed subtler shape changes and stronger sexual dimorphism. The findings highlight how anthropogenic landscape modification can act as a selective force on dispersal-related traits.

Urbanization represents another axis of environmental pressure, explored by Piccinali et al., who analyzed morphological variation in *T. infestans* populations from urban and rural areas of San Juan, Argentina. Their results showed consistent size reductions in urban populations, accompanied by shape modifications in wings and pronota. These patterns align with the “simplification hypothesis,” positing that urban environments select for smaller, less complex morphologies. The study underscores how cities are reshaping vector evolution, demanding tailored surveillance and control strategies for urban Chagas disease.

Expanding to a macroecological scale, Verly et al. examined phenotypic variation across populations of *T. garciabesi* spanning Argentina and Paraguay. They identified associations between flight-related morphology and climatic, geographic and vegetation variables, revealing isolation by distance and environmentally structured variation. This study highlights the power of geometric morphometrics to link environmental gradients with functional morphological traits.

Finally, hybridization was the focus of Lara et al., who compared wing morphometrics of *Panstrongylus chinai*, *P. howardi*, and their hybrids in the Southern Andean and the Central Coastal regions of Ecuador. The study found that hybrids exhibited intermediate traits and smaller wing sizes, suggesting reduced fitness. Shape analyses revealed hybrid morphologies resembling one parent or the other depending on cross direction and sex. These findings indicate that hybridization can generate phenotypic variation with possible functional consequences for dispersal and fitness.

## Not only a vector-borne disease hotspot, but also a source of knowledge and resilience

The collective message emerging from this Research Topic is clear: the Global South is not merely a region affected by vector-borne diseases—it is also a source of research and discovery in vector biology. By combining nucleic acid quantification, DNA barcoding, morphometric analyses, and ecological information, the studies presented here highlight the ability of Global South countries to generate high-quality science that, adjusted to local challenges, adds a unique and valuable perspective to global research.

Future challenges will require sustaining and expanding this integrative vision. As climate change, urban expansion, and globalization continue to reshape vector biology, collaborative research combining genetic, ecological, and evolutionary approaches will be essential to anticipate and mitigate disease emergence. Building stronger bridges between science, decision-makers, and affected communities will be essential. Such connections will enable responses not only technically sound but also socially and culturally grounded, fostering more equitable and effective strategies to anticipate and mitigate disease emergence.
